# Supplementary material for: Dim light at night impacts circadian rhythms and Alzheimer’s disease-like neuroinflammation and neuropathology in humanized APP SAA knock-in mice
Source: Sleep. 2026 Feb 20;49(6):zsag041. doi: 10.1093/sleep/zsag041 (PMC13266548; doi:10.1093/sleep/zsag041)
Supplement: Response_to_Reviewers_zsag041 [file response_to_reviewers_zsag041.docx]

**Reviewer 1**

**Reviewer 1:** *“There were issues with the claims about CD45 and MHCII localization: “In contrast to CD45, which was predominantly plaque-associated, MHCII labeling did not map specifically to areas of plaques…” and “Whereas CD45 remained tightly plaque-associated, MHCII was more diffusely distributed…” However, Figures 4 and 5 do not include co-staining for amyloid plaques, preventing colocalization from being determined. To support this claim, CD45 and MHCII labeling should be combined with the Aβ staining shown in Figure 2 using fluorescent secondary antibodies to confirm plaque association.”*

**Response:** To address this concern, we performed new multiplex immunofluorescence experiments combining IBA1 with either CD45 or MHCII, together with Amylo-Glo to label Aβ plaques. Using HALO Object Colocalization FL, we quantified plaque-associated (Amylo-Glo⁺) and non-plaque-associated (Amylo-Glo⁻) IBA1⁺ microglia expressing each marker. The results were in strong agreement with our interpretation of the IHC findings: CD45⁺ cells were largely plaque-associated, while the dLAN-associated increase in MHCII⁺ cells was not specifically plaque-associated. We have revised the methods, results, and added a new figure and figure legend. As the findings are consistent with our earlier observations, no changes to the discussion were needed. For convenience, we include the figure and figure legend here:

***Figure 6. Dim light at night differentially increases CD45⁺ and MHCII⁺ microglial populations in hAPPSAA KI mice.***

***(A)*** *Low-magnification multiplex immunofluorescence images showing SYTOX nuclear stain, Amylo-Glo–labeled Aβ plaques, IBA1⁺ microglia, and CD45⁺ cells in hAPPSAA KI mice maintained under a standard 12/12 light–dark cycle (LD) or dim light at night (dLAN). Magenta arrows indicate the regions shown at higher magnification.* ***(B)*** *Low-magnification images showing SYTOX, Amylo-Glo, MHCII⁺ cells, and CD45⁺ cells in the same conditions, with arrows indicating corresponding high-magnification fields. For both A and B, higher-magnification panels show individual fluorescence channels overlaid with SYTOX, followed by merged images.* ***(C–D)*** *Quantification of non–Aβ-plaque–associated microglia (Aβ⁻) expressing CD45 (C) or MHCII (D), reported as cells per mm².* ***(E-F)*** *Quantification of Aβ-plaque–associated microglia (Aβ⁺) expressing* *CD45* ***(E)*** *or MHCII* ***(F)****, reported as cells per mm². Symbols represent individual mice; circles denote females and squares denote males; bars show mean ± SEM. Sample sizes were: hAPPWT KI-LD (n = 7, 4F/3M), hAPPWT KI-dLAN (n = 12, 6F/6M), hAPPSAA KI-LD (n = 12, 7F/5M), hAPPSAA KI-dLAN (n = 8, 4F/4M). Analyses were conducted using two-way ANOVA (genotype × light) with planned within-genotype contrasts where appropriate. Main effects and interactions are indicated below the panels. Asterisks denote significance levels (*p < 0.05, **p < 0.01, ***p < 0.001).*

**Reviewer 1:** *“Similarly, the authors note that “CD45 can also be detected on lymphocytes, although these were not commonly observed.” However, this statement is not supported by any figure or quantification. Either an analysis confirming the rarity of lymphocytes should be added or external justification provided (e.g., from literature), as the current evidence is insufficient.”*

**Response:** We had not previously included quantitative data to support this statement. To address this, we quantified lymphocyte-like cells using a HALO-AI classifier trained to distinguish lymphocyte morphology from non-lymphocytic CD45⁺ cells. As now reported in the Results, “*Quantification using a HALO-AI classifier trained to distinguish lymphocyte morphology revealed few lymphocyte-like cells in both neocortex (2.06–2.79 cells/mm², SD 0.69–0.80) and hippocampus (2.47–2.94 cells/mm², SD 0.85–1.43), and these counts did not differ by genotype or dim light at night exposure.*” These data confirm that lymphocytes were rare across regions and conditions and support the conclusion that the observed increases in CD45 labeling primarily reflect non-lymphocytic cell populations.

***Reviewer 1:*** *“Figures 4 and 5 rely on Halo AI to quantify CD45+ and MHCII+ cells. Based on the example images, adjacent cells appear to be merged during analysis. More detail on Halo AI training parameters is needed... Furthermore, since the conclusions are centered on microglial activation, co-labeling for Iba-1 would substantially strengthen these analyses. Quantifying CD45 and MHCII expression within Iba-1+ cells would allow the authors to distinguish between increased activation versus increased microglial numbers overall.”*

**Response:** The reviewer is correct that adjacent CD45⁺ and MHCII⁺ profiles are frequently segmented as contiguous objects during analysis. This outcome reflects the density of immunoreactive profiles in hAPPSAA KI tissue and the inherent limitations of DAB-based segmentation. The HALO-AI classifier was trained to distinguish DAB-positive signal from background, not to separate individual cells or enforce object splitting. We have added the following detail to the Methods: “*The HALO-AI classifier used to segment CD45⁺ and MHCII⁺ immunoreactive clusters was trained on 58 manually produced glial cluster annotations across 5 manually defined regions spanning all genotypes and light conditions, using 6470 iterations, and achieved an entropy score of 0.092. Markup overlays were visually reviewed across all slides to confirm segmentation accuracy prior to batch analysis.*” “*A separate HALO-AI classifier trained to identify lymphocyte-like morphology was developed using 25 manually produced lymphocyte annotations, 62 manually defined areas, 42,720 iterations, and achieved an entropy score of 0.111. Markups were visually validated for accuracy prior to quantification. All analyses were performed blind to genotype and light conditions.*” As a result, the quantified units represent clusters of immunoreactivity rather than individual cells, which is why these outcomes are consistently referred to as clusters throughout the manuscript and figure legends. Cluster size and number were defined objectively based on the area of segmented staining and analyzed using quartile-derived size bins, as detailed in the Methods. Importantly, in regions of dense microgliosis, even high-resolution confocal microscopy often does not reliably resolve individual cells due to overlapping processes, tight packing, or multinucleated morphology. Distinguishing whether such structures represent multiple apposed cells versus complex or multinucleated microglia is beyond the scope of the current study. We therefore focused on cluster-based metrics as a robust and biologically meaningful measure of regional immune remodeling rather than attempting to infer individual cell counts where reliable segmentation is not feasible.

To directly address the reviewer's second point regarding microglial identity and activation state, we have added new multiplex immunofluorescence data in Figure 6, which explicitly co-labels IBA1 with CD45 or MHCII and Amylo-Glo. These analyses quantify CD45⁺ and MHCII⁺ expression specifically within IBA1⁺ microglial populations and distinguish plaque-associated from non–plaque-associated microglia. This additional dataset confirms that the observed changes reflect altered microglial phenotypes and spatial organization rather than nonspecific increases in immune cell numbers. Together, the cluster-based DAB analyses (Figures 4–5) and the IBA1-resolved immunofluorescence analyses (Figure 6) provide complementary and internally consistent evidence supporting our conclusions.

**Reviewer 1:** *“The authors noted that “dim light at night impaired rhythms similarly in hAPPWT and hAPPSAA mice.” However, Figure 1C shows a main effect of genotype on intradaily variability. This discrepancy should be acknowledged and the interpretation tempered or revised accordingly.”*

**Response:** We thank the reviewer for catching this discrepancy. The reviewer is correct that a main effect of genotype was observed for intradaily variability. However, this effect reflects higher baseline rhythm fragmentation in hAPPSAA KI mice collapsed across light conditions, not a differential response to dim light at night. The absence of a genotype × light interaction confirms that both genotypes responded similarly to dim light at night. We have revised the results text to accurately report the genotype main effect while clarifying this distinction. Specifically, we have added: “*There were no genotype × light interactions for any circadian parameter, indicating that dim light at night impaired rhythms similarly in hAPP^WT^ KI and hAPP^SAA^ KI mice. A main effect of genotype was observed for intradaily variability (****Fig. 1C****; F(1,35) = 6.18, p = 0.018), reflecting higher rhythm fragmentation in hAPP^SAA^ KI mice collapsed across light conditions.*”

**Reviewer 1:** *“Mice were euthanized at ZT7–8, yet most dLAN-associated differences occurred during the dark phase. Please justify this timepoint selection or note its limitations.”*

**Response:** We have added the following clarification to the Methods: “*This timepoint was selected to correspond to the mid-light (rest) phase, minimizing variability associated with peak locomotor activity and avoiding potential confounds from acute activity-induced inflammatory signaling that can occur during the dark (active) phase.*”

**Reviewer 1:** *“Terms such as ‘activated,’ ‘reactive,’ and ‘primed’ are used interchangeably in describing microglia. Consistent terminology—or clear definitions for each—would aid interpretation.”*

**Response:** We thank the reviewer for raising this point. Upon review, each term was used with distinct intent. ‘Reactive’ was used to describe CD45⁺ microglia, reflecting the established use of CD45 upregulation as a marker of microglial reactivity. ‘Primed’ was used specifically to describe MHCII⁺ microglia; we have expanded this section to define the term: “*MHCII, a key antigen-presenting molecule, is induced by interferons and is often used as a marker of primed microglia. These MHCII-expressing cells have shifted toward a heightened baseline inflammatory state without active cytokine production, yet mount exaggerated responses to subsequent stimuli and may represent a form of trained immunity (Wangler & Godbout, 2023).*” ‘Activated’ appeared once in the introduction in a general context. We agree this term was imprecise and have revised the sentence from “neuroinflammation mediated by activated microglia” to “*neuroinflammation mediated by microglia*.”

**Reviewer 1:** *“In figures after Figure 1, circles and squares appear to represent different sexes but are never defined... Data points are also large and overlapping... Add a legend or key indicating what each color/label represents...”*

**Response:** We have added a graphical legend to Figures 2, 3, and 6 indicating that circles denote females, squares denote males, and color distinguishes LD (open) from dLAN (filled). For Figures 4, 5, and 7, space constraints precluded an embedded legend; this information is provided in the figure legend text. Regarding data point size, we have balanced visibility of individual values against overlap; reducing symbol size further would make sex distinctions difficult to discern, particularly in print.

**Reviewer 1:** *“In Figure 2, the authors repeatedly display that hAPPWT mice do not produce plaques. This could be shown in one subfigure of Figure 2 and cut from the others... In Figure 2, the graphs for different plaque densities don’t add anything to the interpretation and just add to clutter. These could be made into a supplementary figure.”*

**Response:** We appreciate the reviewer's attention to figure clarity. In response, we have revised Figure 2 to remove hAPPWT KI data from the plaque density graphs (panels E and G), as these animals do not develop plaques and the zero values added no information. The plaque density data for hAPPSAA KI mice has been retained in the main figure. While the absence of significant differences between light conditions may appear to be a negative finding, this result is scientifically meaningful. Specifically, it demonstrates that dim light at night does not alter the morphological composition of Aβ deposits (i.e., the ratio of diffuse to dense-core plaques). A shift from diffuse to compact plaques, or vice versa, would suggest changes in plaque maturation or microglial compaction activity, which has been reported in other models of immune modulation. The absence of such a shift, despite increased overall plaque burden, indicates that dLAN-associated increases in Aβ pathology reflect a general acceleration of deposition rather than altered plaque processing. We believe retaining this data in the main figure strengthens the interpretation by showing what did not change alongside what did. Regarding the representative images, we have retained hAPPWT KI panels to provide a visual reference for the absence of plaque pathology in control animals, which aids readers in interpreting the hAPPSAA KI data.

Regarding the suggestion to analyze only hAPPSAA KI mice for plaque outcomes, we have retained the interaction analysis because hAPPWT KI mice carry a humanized Aβ sequence and produce soluble Aβ, as demonstrated by the PBS ELISA data. These mice have the molecular capacity to form plaques under appropriate conditions. The absence of plaque formation in hAPPWT KI mice exposed to dLAN is therefore informative rather than trivial, demonstrating that dim light at night does not independently induce Aβ aggregation but rather exacerbates pathology in a mutation-prone background.

**Reviewer 1:** *“In Figure 2, the authors never explain the relevance of PBS vs formic acid soluble Aβ. A sentence or two in the results or introduction would help clear this up.”*

**Response:** We have added the following sentence to the Results section to clarify the relevance of these two extraction methods: “*To distinguish soluble from deposited Aβ pools, we performed sequential biochemical extraction using PBS (which recovers soluble Aβ species including monomers and oligomers) followed by formic acid (which solubilizes fibrillar, plaque-associated Aβ).”*

**Reviewer 1:** *“In Figure 3, aligning M and N with the row of images that they are analyzing would improve clarity (same with Figure 4).”*

**Response:** We appreciate this suggestion for improving figure clarity. We have revised Figures 3 and 4 to align the quantification graphs with their corresponding image rows to the best of our ability given space constraints. We have also added a graphical legend to Figure 3 indicating sex and light condition. Panel labels and figure legends clearly indicate which graphs correspond to which images.

**Reviewer 1:** *“Figure 6 is titled “Cortical and hippocampal cytokines and chemokines are elevated in hAPPSAA KI mice, with minimal effects of dim light at night”. dLAN didn’t show a significant effect on any cytokine or chemokine, So this title could just be limited to “Cortical and hippocampal cytokines and chemokines are elevated in hAPPSAA KI mice”*

**Response:** We thank the reviewer for this suggestion. We have revised the result section title to “*Cortical and hippocampal cytokines and chemokines are elevated in hAPPSAA KI mice*” , as suggested.

**Reviewer 1:** *“The authors write “There is also evidence that this exaggerated cytokine response is sex dependent, with females showing a greater effect” but note that they saw no sex differences. Could that graph be displayed as a supplementary graph?”*

**Response:** We appreciate the reviewer’s comment and agree that this point warrants clarification. The statement regarding sex-dependent exaggeration of cytokine responses refers to prior work (Chen et al., 2021) demonstrating enhanced cytokine priming in females following dim light at night exposure under immune challenge, not to effects observed in the present study. In our dataset, we did not detect sex differences in cytokine expression. Sex is already indicated in the plotted data using distinct symbols. Plotting males and females separately in a supplemental figure invites overinterpretation of null effects. Therefore, we have not included this graph as supplemental data. We have revised the discussion to make this distinction explicit, as follows: “There is also evidence *from prior studies* that exaggerated cytokine responses to dim light at night are sex dependent, with females showing a greater effect under immune challenge conditions.”

**Reviewer 2**

**Reviewer 2:** *“What is the novel contribution of this study? The relationship between circadian disruption and AD pathology progression has been extensively documented. The use of dim light at night to induce circadian disruption... appears to be primarily a methodological variation rather than a conceptual advance. While the humanized APP knock-in models are relatively newer... how does this fundamentally change our understanding?”*

**Response:** We view dim light at night not merely as an experimental manipulation, but as a prevalent environmental condition that affects large segments of the population. While epidemiological studies have revealed that dim light at night is statistically associated with higher incidence of MCI and AD, correlation does not prove causation. To address causation and explore underlying mechanisms, this study investigated the effects of dLAN on neuroinflammation and amyloidosis in an animal model. Importantly, previous dLAN studies have not examined amyloid pathology, and no prior work has used humanized APP knock-in models that express physiological levels of Aβ without overexpression artifacts. The inclusion of hAPPWT KI mice, which carry humanized Aβ and produce soluble Aβ but lack disease-linked mutations, allowed us to demonstrate that dLAN does not independently induce Aβ aggregation but rather exacerbates pathology in a mutation-prone background. This distinction would not be possible in traditional transgenic overexpression models. We note that while dLAN disrupts circadian rhythms, and circadian rhythm disruption is associated with AD, we did not directly test whether the circadian rhythm disruption itself is responsible for the effects of dLAN on AD neuropathology. Light has a variety of visual and nonvisual effects that could potentially contribute to the effects of dLAN on AD neuropathology.

**Reviewer 2:** *“The single 8-week endpoint severely limits interpretation of the temporal relationships between circadian disruption, inflammation, and pathology... Without temporal profiling, these possibilities remain indistinguishable, significantly weakening any novel mechanistic insights.”*

**Response:** We agree that a single timepoint does not permit definitive conclusions about the temporal ordering of circadian disruption, neuroinflammation, and amyloid pathology. This limitation is acknowledged in our Discussion: “*Pathological analyses were performed at a single timepoint, limiting conclusions about the temporal progression of immune and amyloid changes.*” We also note that “*Future studies could measure plaque density and morphology across timepoints to assess whether changes in microglial phenotype correspond to structural alterations*.” The primary aim of this study was to establish whether chronic dim light at night exacerbates AD-related pathology in the hAPPSAA KI model, which our data support. Temporal profiling to dissect mechanistic sequence represents an important next step that is now justified by these findings.

**Reviewer 2:** *“The biological significance of the amyloid pathology changes is questionable without functional assessments. The increases in hippocampal plaque burden and neocortical soluble Aβ are statistically significant but appear modest... Without behavioral or cognitive testing, it's impossible to determine if these changes are meaningful.”*

**Response:** We acknowledge that behavioral and cognitive assessments were not included in this study, a limitation we address in the Discussion: “*In addition, cognitive behavior and synaptic physiology were not assessed, so the functional consequences of dim light at night exposure remain uncertain.*” However, we respectfully disagree that statistically significant increases in amyloid pathology require concurrent behavioral deficits to be considered biologically meaningful. Aβ accumulation is a defining feature of AD and a validated target for disease-modifying therapies; interventions that reduce plaque burden are pursued clinically regardless of immediate cognitive correlates. Furthermore, pathological changes often precede detectable behavioral deficits in mouse models, particularly at the age examined here. The purpose of this study was to determine whether environmental circadian disruption exacerbates AD-related neuropathology, which our data support. Functional consequences of these pathological changes represent an important future direction but are beyond the scope of this initial characterization.

**Reviewer 2:** *“The authors state that “sex was included as a factor in initial models, but no main effects or interactions were detected; data were therefore pooled across sexes” ... yet they provide no supporting data for this crucial decision... The authors must show the actual sex-stratified data for key outcomes... Furthermore, were estrous cycles monitored in females? At 12-13 months, were all females in persistent diestrus/anestrus, which might explain the lack of differences?”*

**Response:** We fully appreciate the reviewer’s concern regarding sex as a biological variable. We did not intend to dismiss sex differences, but rather to report the statistical outcomes of our specific dataset. Sex is displayed in Figures 2–7 using distinct symbols (circles for females, squares for males), allowing readers to visually assess whether patterns differ between sexes for any outcome. We have added a graphical legend to Figures 2, 3, and 6 clarifying these symbols; for Figures 4, 5, and 7, this information is provided in the figure legend text. We included sex as a factor in our statistical models and found no significant main effects or interactions; pooling across sex was therefore justified and follows standard practice when sex effects are absent. We have added clarification to the Methods: “*The study was not designed or powered to detect sex-specific effects, and the absence of significant sex interactions should not be taken as definitive evidence of no effect.*” Providing a supplemental table of non-significant sex effects for every endpoint would be lengthy and would not alter interpretation, though we are willing to include this if the editor feels it is necessary.

Regarding estrous cycle monitoring: as stated in the Methods, the mice in this study were 12–13 months of age, which is past reproductive senescence in C57BL/6 mice (typically occurring around 9–12 months). At this age, females are generally in persistent diestrus or anestrus, and estrous monitoring would not be expected to reveal meaningful variation.

**Reviewer 2:** *“The observation of differential spatial distribution between MHCII+ and CD45+ microglia... is potentially the most novel finding, yet it remains underdeveloped... Is this spatial pattern unique to dLAN exposure, or would any circadian disruption method produce similar results?”*

**Response:** We appreciate the reviewer's recognition that the differential spatial distribution of MHCII⁺ and CD45⁺ microglia is a potentially novel finding. In response to comments from both Reviewer 1 and Reviewer 2 (see Reviewer 1 response for details), we have now substantially developed this observation with new multiplex immunofluorescence experiments (Figure 6) that co-label IBA1 with CD45 or MHCII together with Amylo-Glo to mark Aβ plaques. These analyses quantify plaque-associated versus non-plaque-associated microglia expressing each marker and confirm that CD45⁺ cells are largely plaque-associated while the dLAN-associated increase in MHCII⁺ microglia is not specifically plaque-associated. This provides direct quantitative support for our interpretation.

Whether this spatial pattern is unique to dim light at night or would be observed with other forms of circadian disruption is an important question. We have added the following to the Discussion: “*Whether this spatial pattern is unique to dim light at night or would be observed with other forms of circadian disruption such as phase shifts, constant light, or sleep fragmentation remains to be determined; to our knowledge, microglial spatial distribution relative to plaques has not been examined following other circadian disruption paradigms.*” This represents a valuable future direction that could help determine whether the effects we observe are specific to light-mediated circadian disruption or reflect a more general consequence of circadian or sleep disturbance.
